# Delivery of *Mycobacterium tuberculosis* epitopes by *Bordetella pertussis* adenylate cyclase toxoid expands HLA-E-restricted cytotoxic CD8^+^ T cells

**DOI:** 10.3389/fimmu.2023.1289212

**Published:** 2023-12-01

**Authors:** Giusto D. Badami, Marco P. La Manna, Paola Di Carlo, Ondrej Stanek, Irena Linhartova, Nadia Caccamo, Peter Sebo, Francesco Dieli

**Affiliations:** ^1^ Central Laboratory of Advanced Diagnosis and Biomedical Research (CLADIBIOR), AOUP Paolo Giaccone, Palermo, Italy; ^2^ Department of Biomedicine, Neuroscience and Advanced Diagnosis (BIND), University of Palermo, Palermo, Italy; ^3^ Department of Sciences for Health Promotion and Mother-Child Care “G. D’Alessandro”, University of Palermo, Palermo, Italy; ^4^ Laboratory of Molecular Biology of Bacterial Pathogen, Institute of Microbiology of the Czech Academy of Sciences, Prague, Czechia

**Keywords:** *Mycobacterium tuberculosis*, vaccine, immunotherapy, *Bordetella pertussis* adenylate cyclase, cytotoxic t lymphocytes, HLA-E, peptides

## Abstract

**Introduction:**

Tuberculosis (TB) remains the first cause of death from infection caused by a bacterial pathogen. Chemotherapy does not eradicate *Mycobacterium tuberculosis* (Mtb) from human lungs, and the pathogen causes a latent tuberculosis infection that cannot be prevented by the currently available Bacille Calmette Guerin (BCG) vaccine, which is ineffective in the prevention of pulmonary TB in adults. HLA-E-restricted CD8+ T lymphocytes are essential players in protective immune responses against Mtb. Hence, expanding this population *in vivo* or *ex vivo* may be crucial for vaccination or immunotherapy against TB.

**Methods:**

The enzymatically inactive Bordetella pertussis adenylate cyclase (CyaA) toxoid is an effective tool for delivering peptide epitopes into the cytosol of antigen-presenting cells (APC) for presentation and stimulation of specific CD8+ T-cell responses. In this study, we have investigated the capacity of the CyaA toxoid to deliver Mtb epitopes known to bind HLA-E for the expansion of human CD8+ T cells *in vitro*.

**Results:**

Our results show that the CyaA-toxoid containing five HLA-E-restricted Mtb epitopes causes significant expansion of HLA-E-restricted antigen-specific CD8+ T cells, which produce IFN-γ and exert significant cytotoxic activity towards peptide-pulsed macrophages.

**Discussion:**

HLA-E represents a promising platform for the development of new vaccines; our study indicates that the CyaA construct represents a suitable delivery system of the HLA-E-binding Mtb epitopes for *ex vivo* and *in vitro* expansion of HLA-E-restricted CD8+ T cells inducing a predominant Tc1 cytokine profile with a significant increase of IFN-γ production, for prophylactic and immunotherapeutic applications against Mtb.

## Introduction

The World Health Organization (WHO) Global Tuberculosis Control Report indicates that globally, 10.6 million people fell ill with TB disease in 2021. Moreover, it was estimated that one-fourth of the global population is latently infected with Mtb (1). The 6.7% of all TB cases were among HIV-positive individuals. The highest percentages of TB cases were reported in South-East Asia (45%), Africa (23%), and the Western Pacific (18%), while the Eastern Mediterranean (8.1%), the Americas (2.9%), and Europe (2.2%) reported the lowest percentages. Between 2005 and 2019, there were fewer TB-related deaths worldwide each year, but in 2020 and 2021, the trend was the opposite.

Of 1.6 million deaths in 2021, 1.4 million were among HIV-negative persons and 187,000 among HIV-positive people. Globally, there were an estimated 450,000 MDR/RR-TB incidents in 2021, with a 42% mortality rate.

Active TB is curable with chemotherapy, but despite drug treatment, patients may not develop sterilizing immunity (2–4). Moreover, the BCG vaccine can prevent disseminated forms of TB in children but fails to protect adults from pulmonary TB (5). This scenario raises an urgent need to design the most effective TB vaccine.

CD8^+^ T cells are critical players in cell-mediated immunity and contribute to protection against intracellular pathogens by interferon (IFN)-γ production and by eliminating both infected cells and intracellular pathogens by cytotoxic activity (6, 7). In humans, Mtb-specific CD8^+^ T cells recognise epitopes presented by both major histocompatibility complex (MHC) class Ia (HLA-A, -B and -C) and (8, 9) and MHC class Ib (HLA-E, MR1) and CD1 molecules (10).

HLA-E is generally acknowledged for preventing NK lysis upon binding to NKG2A/CD94 complex (11, 12), but it can present epitopes to CD8^+^ T cells, HLA-E has e very low polymorphism (10, 13) and, relevant to Mtb-specific responses, is enriched in Mtb phagosomes, thus potentially binding a vast array of Mtb peptides (7, 14). Moreover, and different than MHC class Ia molecules, HLA-E is resistant to HIV Nef-mediated downregulation, rendering HLA-E a good candidate as an antigen-presenting molecule for the formulation of a peptide-based subunit vaccine for immunotherapy in infectious diseases or tumour immunology (7, 15, 16). Accordingly, Almond et al. demonstrated that virus-specific HLA-E restricted CD8^+^ T cells protected macaques from Simian immunodeficiency virus (SIV) after vaccination with a cytomegalovirus SIV-gag protein model (17). Finally, HLA-E-restricted CD8^+^ T cells were efficient in killing macrophages infected with virulent Mtb alone or co-infected with Mtb and HIV-1 and in reducing the viability of both pathogens (7).

Recently, *Bordetella pertussis* CyaA has been established as a promising tool for the delivery of antigens for processing and presentation by dendritic cells (DCs) to CD8^+^ T cells (18–20). In particular, CyaA-AC (–) toxoid, thanks to its pore-forming activity, triggers Toll-like receptors and inflammasome signaling-independent maturation of CD11b-expressing DCs (18). Indeed, these CyaA constructs induced antigen-specific CD8^+^ T cells responding against pathogens (HIV, CMV, LCMV, HPV or influenza) (21), bacteria such as Mtb, and parasites such as *Plasmodium berghei* (22, 23 *).*, melanoma or HPV-induced tumoral cells (16, 24, 25). Therefore, the efficacy of cGMP preparations of CyaA toxoids has been evaluated under clinical trials in humans for treating metastatic melanoma and papillomavirus-induced cervical cancer. The CyaA toxoid possesses aside induction of T cell immune responses as described above, also an inherent adjuvant capacity (18) and, most importantly, can shift the immune response from Th2 to a mixed Th1/Th2 type (26, 27). Here, we used five different Mtb epitopes, for which we previously demonstrated binding to HLA-E molecules and presentation to HLA-E-restricted CD8^+^ T cells. CyaA toxoids delivering these epitopes were evaluated for their ability to induce human CD8^+^ T cell responses *in vitro*.

## Materials and methods

### Human blood samples

Peripheral blood was obtained from 42 adults, 20 with LTBI, (age range 35-60 yrs), 12 with active TB disease (6 females, six males, age range 20-40 yrs), and ten healthy donors (6 females and four males, age range 30 -52 yrs) (see **Table 1**). All the subjects were enrolled by clinicians at the Dipartimento di Medicina Clinica e delle Patologie Emergenti, University Hospital, Palermo, Italy. All TB patients recruited in the study showed typical clinical and radiological symptoms compatible with active pulmonary TB, and Mtb was isolated bacteriologically to confirm the diagnosis. None of the TB patients had received BCG vaccination or had been treated with immunosuppressive or anti-tubercular medications, including steroids. Healthcare professionals who had a positive PPD skin test but showed no symptoms or signs of active TB were classified as having latent tuberculosis infection (LTBI). The diagnosis of TB infection was confirmed using interferon IFN-γ release assays (IGRAs) Quantiferon-TB Plus (QFT-Plus). All participants provided written informed consent, and the study was approved by the University Hospital Ethical Committee in accordance with the principles of the Helsinki Declaration and “Good Clinical Practices”.

**Table 1:** Characteristics of TB patients, LTBI, and HD recruited in the study.

Variable	HD	TB	LTBI
**Total subjects, n**	10	12	20
**Age (range)**	30-52	20-40	25-60
**Clinical characteristics**	a)	Pulmonary TB (9), Extra-pulmonary TB (3)	Healthy, Mantoux positive
**Nationality**	b)	Italian (4) North African (8)	Italian (5) North African (15)

a) No information about the anonymous samples was available.

b) Samples were obtained from anonymous blood bank donors.

Peripheral blood mononuclear cells (PBMC) were isolated from heparinized blood samples by Ficoll-Hypaque (Sigma) density gradient centrifugation (750g for 20 min.). PBMCs were then collected, washed twice (500×g for 5 min) in RPMI 1640 medium (Euroclone, Pero, Italy) supplemented with HEPES 20 mM, penicillin 100 U/mL and streptomycin 100 μg/mL, and were resuspended in 1 ml of complete RPMI medium (10% heat-inactivated FCS, L-glutamine 2 mM). PBMCs that were not immediately used were frozen by adding an equal volume of FCS 20%/DMSO solution to the complete medium in which the cells were resuspended. The frozen PBMCs were stored in liquid nitrogen. When needed, PBMCs were thawed at 37°C and immediately put in pre-warmed (37°C) FCS, centrifuged for 5 min at low speed (250g), resuspended in complete RPMI medium and counted with trypan blu dye. The PBMCs were rested for one hour in an incubator at 37°C with 5% CO_2_ and counted before experiments.

### Construction and purification of recombinant adenylate cyclase CyaA toxoids carrying HLA-E-restricted Mtb epitopes

The initial CyaAM8a toxoid used for the construction of CyaA-LPE and CyaA-SPE toxoids with inserted HLA-E-restricted Mtb epitopes for delivery into APCs, was prepared by modifying the adenylate cyclase toxoid B+D18 described previously (21). Briefly, the CyaAM8a toxoid has a Δ3–370 deletion of residues 3 to 370 of the AC domain of CyaA. The missing AC polypeptide segment was replaced by heterologous polypeptide sequences comprising the oligo epitopes of choice. First, the overlapping CD8^+^ and CD4^+^ T cell epitopes for MHC class I and class II presentation of the peptides derived from chicken egg ovalbumin (OVA), comprising residues 257 to 276 of OVA and flanked by the natural flanking sequences (leqleSIINFEKLTEWTSSNVMEERkikvylpr) were introduced. This control mock toxoid is labelled further throughout the paper as CyaA. Next, Mtb antigenic oligo epitope sequences were inserted, flanked by pairs of arginine residues. The inserted Mtb oligo epitope comprised only the selected p62 and p68 epitopes in the shorter construct (CyaA-SPE), or all five HLA-E-restricted epitopes p34, p44, p55, p62, and p68 in the longer construct (CyaA-LPE). The entire amino acid sequences of the CyaA-SPE and CyaA-LPE constructs are listed in **Table 2**, with the OVA-derived reporter epitopes sequence highlighted in blue and bold type and the Mtb-derived oligo epitope antigenic sequences in red type. All plasmid constructs were sequence-verified by DNA sequencing before use for the production of the CyaA-SPE and CyaA-LPE toxoids in *E. coli* BL21/pMM100 (*lacI^q^*) cells and purification of the toxoids by the procedure described previously (21, 28), as shown in **Supplementary Figure S1A**.

**Table 2:** Characteristics of recombinant adenylate cyclase CyaA-SPE and CyaA-LPE toxoids carrying HLA-E-restricted *M. tuberculosis* epitopes.

Construct	Num. of amino acids	Molecular weight	Theoretical pI
CyaA-SPE	1418	147129,8	4.49
Sequence	MGTVNSLEQLE**SIINFEKLTEWTSSNVMEER**KIKVYLPRARRRTGFGITRMPPLGHELRPEVARVLRPGGHFLYTDSTSRRPSRSKFSPDVLETVPASPGLRRPSLGAVERQDSGYDSLDGVGSRSFSLGEVSDMAAVEAAELEM TRQVLHAGARQDDAEPGVSGASAHWGQRALQGAQAVAAAQRLVHAIALMTQFGRAGSTNTPQEAASLSAAVFGLGEASSAVAETVSGFFRGSSRWAGGFGVAGGAMALGGGIAAAVGAGMSLTDDAPAGQKAAAGA EIALQLTGGTVELASSIALALAAARGVTSGLQVAGASAGAAAGALAAALSPMEIYGLVQQSHYADQLDKLAQESSAYGYEGDALLAQLYRDKTAAEGAVAGVSAVLSTVGAAVSIAAAASVVGAPVAVVTSLLTGALNGIL RGVQQPIIEKLANDYARKIDELGGPQAYFEKNLQARHEQLANSDGLRKMLADLQAGWNASSVIGVQTTEISKSALELAAITGNADNLKSVDVFVDRFVQGERVAGQPVVLDVAAGGIDIASRKGERPALTFITPLAAPGEEQ RRRTKTGKSEFTTFVEIVGKQDRWRIRDGAADTTIDLAKVVSQLVDANGVLKHSIKLDVIGGDGDDVVLANASRIHYDGGAGTNTVSYAALGRQDSITVSADGERFNVRKQLNNANVYREGVATQTTAYGKRTENVQYR HVELARVGQVVEVDTLEHVQHIIGGAGNDSITGNAHDNFLAGGSGDDRLDGGAGNDTLVGGEGQNTVIGGAGDDVFLQDLGVWSNQLDGGAGVDTVKYNVHQPSEERLERMGDTGIHADLQKGTVEKWPALNLFS VDHVKNIENLHGSRLNDRIAGDDQDNELWGHDGNDTIRGRGGDDILRGGLGLDTLYGEDGNDIFLQDDETVSDDIDGGAGLDTVDYSAMIHPGRIVAPHEYGFGIEADLSREWVRKASALGVDYYDNVRNVENVIGTSM KDVLIGDAQANTLMGQGGDDTVRGGDGDDLLFGGDGNDMLYGDAGNDTLYGGLGDDTLEGGAGNDWFGQTQAREHDVLRGGDGVDTVDYSQTGAHAGIAAGRIGLGILADLGAGRVDKLGEAGSSAYDTVSGIENV VGTELADRITGDAQANVLRGAGGADVLAGGEGDDVLLGGDGDDQLSGDAGRDRLYGEAGDDWFFQDAANAGNLLDGGDGRDTVDFSGPGRGLDAGAKGVFLSLGKGFASLMDEPETSNVLRNIENAVGSARDDVLIGD AGANVLNGLAGNDVLSGGAGDDVLLGDEGSDLLSGDAGNDDLFGGQGDDTYLFGVGYGHDTIYESGGGHDTIRINAGADQLWFARQGNDLEIRILGTDDALTVHDWYRDADHRVEIIHAANQAVDQAGIEKLVEAMAQ YPDPGAAAAAPPAARVPDTLMQSLAVNWR		
CyaA-LPE	1469	152845,5	4.55
Sequence	MGTVNSLEQLE**SIINFEKLTEWTSSNVMEER**KIKVYLPRARRRTGGAIHVMTTVLATLPADHRITDRLPAKAPLLELDVETTRVMATRRNVLDRQRFGITRMPPLGHELRPEVARVLRPGGHFLYTDSTSRRPSRSKFSPDVLET VPASPGLRRPSLGAVERQDSGYDSLDGVGSRSFSLGEVSDMAAVEAAELEMTRQVLHAGARQDDAEPGVSGASAHWGQRALQGAQAVAAAQRLVHAIALMTQFGRAGSTNTPQEAASLSAAVFGLGEASSAVAETVSGFFR GSSRWAGGFGVAGGAMALGGGIAAAVGAGMSLTDDAPAGQKAAAGAEIALQLTGGTVELASSIALALAAARGVTSGLQVAGASAGAAAGALAAALSPMEIYGLVQQSHYADQLDKLAQESSAYGYEGDALLAQLYRDKTA AEGAVAGVSAVLSTVGAAVSIAAAASVVGAPVAVVTSLLTGALNGILRGVQQPIIEKLANDYARKIDELGGPQAYFEKNLQARHEQLANSDGLRKMLADLQAGWNASSVIGVQTTEISKSALELAAITGNADNLKSVDVFVDR FVQGERVAGQPVVLDVAAGGIDIASRKGERPALTFITPLAAPGEEQRRRTKTGKSEFTTFVEIVGKQDRWRIRDGAADTTIDLAKVVSQLVDANGVLKHSIKLDVIGGDGDDVVLANASRIHYDGGAGTNTVSYAALGRQDS ITVSADGERFNVRKQLNNANVYREGVATQTTAYGKRTENVQYRHVELARVGQVVEVDTLEHVQHIIGGAGNDSITGNAHDNFLAGGSGDDRLDGGAGNDTLVGGEGQNTVIGGAGDDVFLQDLGVWSNQLDGGAGV DTVKYNVHQPSEERLERMGDTGIHADLQKGTVEKWPALNLFSVDHVKNIENLHGSRLNDRIAGDDQDNELWGHDGNDTIRGRGGDDILRGGLGLDTLYGEDGNDIFLQDDETVSDDIDGGAGLDTVDYSAMIHPGRIVA PHEYGFGIEADLSREWVRKASALGVDYYDNVRNVENVIGTSMKDVLIGDAQANTLMGQGGDDTVRGGDGDDLLFGGDGNDMLYGDAGNDTLYGGLGDDTLEGGAGNDWFGQTQAREHDVLRGGDGVDTVDYSQTG AHAGIAAGRIGLGILADLGAGRVDKLGEAGSSAYDTVSGIENVVGTELADRITGDAQANVLRGAGGADVLAGGEGDDVLLGGDGDDQLSGDAGRDRLYGEAGDDWFFQDAANAGNLLDGGDGRDTVDFSGPGRGLDA GAKGVFLSLGKGFASLMDEPETSNVLRNIENAVGSARDDVLIGDAGANVLNGLAGNDVLSGGAGDDVLLGDEGSDLLSGDAGNDDLFGGQGDDTYLFGVGYGHDTIYESGGGHDTIRINAGADQLWFARQGNDLEIRILG TDDALTVHDWYRDADHRVEIIHAANQAVDQAGIEKLVEAMAQYPDPGAAAAAPPAARVPDTLMQSLAVNWR		

The OVA-derived reporter epitope sequence is highlighted in blue and the Mtb-derived epitope sequences are in red.

Bacterial endotoxin was removed during toxoid purification by ion exchange chromatography by washing the toxoid-loaded DEAE Sepharose resin with ten-bed volumes of 1% Triton X100 washing buffer, as previously described (28). In the last step, the toxoids were eluted from the resin with 8M urea, 50 mM Tris-HCl, pH 8.0, and 200 mM NaCl and stored at -20°C. The endotoxin content in the samples was determined using the LAL assay (Thermo Fisher Scientific n. cat. A39552) and was below 40 EU/mg of purified toxoid protein. Before the antigen delivery assay, the CyaA toxoids were pre-diluted in 50 mM Tris-HCl, pH 8.0, 8M urea, 0.2 mM CaCl_2_ buffer to generate the working toxoid stocks that were subsequently diluted 100-fold into urea-free cell incubation media, yielding a final 80 mM urea concentration that does not interfere with cell integrity or antigen presentation functions. The capacity of the toxoids to deliver the inserted SIINFEKL reporter epitope from OVA into DC cytosol for MHC class I-restricted presentation to OVA-specific CD8^+^ T cells B3Z was assessed by the previously described *in vitro* antigen presentation assay (21, 29). Briefly, DC2.4 cells were incubated with different concentrations of toxoids for 4 hrs. After wash with PBS, the cells were cultured for 18 hrs with the B3Z CD8^+^ T-hybridoma cells that selectively recognize the surface complex H-2K^b^ MHC class I molecules bound to OVA_257-264_ peptide SIINFEKL. The amount of accumulated β-galactosidase enzyme, expressed under the IL-2 promoter control (30), was used to assess the stimulation level of B3Z cells, as shown in **Supplementary Figure S1B**.

### Incubation of human DCs with CyaA toxoids

CD14^+^ monocytes were isolated from PBMCs of healthy human donors using the MagCellect Human CD14^+^ Cell Isolation Kit (R&D Systems, Minneapolis, MN) (31). Briefly, 1x10^6^ CD14^+^ cells/mL were put in culture in Iscove’s modified Dulbecco’s medium (IMDM, Life Technologies, Monza, Italy) added with 10% AB human serum (Euroclone, Milan, Italy) and recombinant IL-4 and GM-CSF (both from R&D Systems and both used at 40 ng/mL final concentration) for ten days at 37°C 5% CO_2,_ to induce differentiation to DCs. DCs were washed and incubated with different concentrations of CyaA-SPE, CyaA-LPE, or Lipopolysaccharide (LPS) from *E. coli* (Sigma-Aldrich, St. Louis, MO) as a control, for 24 hrs at 37°C. At the end of the incubation period, cells were washed and incubated with fluorochrome-conjugate mAbs to CD86 (BV510, Clone IT2.2), HLA-DR (FITC, Clone L243) and CD40 (PE, Clone 5C3), all from BD Biosciences (Franklin Lakes, NJ, USA).

### Preparation of HLA-E tetramers and *ex-vivo* tetramer staining

Mtb peptides, p34 (VMTTVLATL), p44 (RLPAKAPLL), p55 (VMATRRNVL), p62 (RMPPLGHEL) and p68 (VLRPGGHFL) have been extensively described previously to bind to HLA-E and to activate CD8^+^ T cells from Mtb-infected individuals (7, 9, 14). **Table 3** shows the characteristics of the five Mtb epitopes. Corresponding HLA-E tetramers (TMs) were prepared as described in (9, 32). After thawing and counting, PBMCs were left for one hour at 37° C with 5% CO_2_ and 2x10^6^ cells for each experimental condition were stained at 37°C for 15 minutes with HLA-E TMs. The five HLA-E PE-conjugated TMs were merged in the same colour channel. Then, samples were centrifuged in the presence of PBS/0.1% BSA and cells viability was assessed by staining them with a viability marker (Vivid fixable violet dye, Life Technologies) and subsequently stained at 4°C for 30 min with fluorochrome-conjugated antibodies to human CD3 (PerCp-Cy5.5, Miltenyi Biotec, Bergisch Gladbach, Germany, Clone REA613) and CD8 (PE-Cy7, Miltenyi Biotec, Clone BW135/80) (33). The cells were washed twice in PBS/0.1% BSA and acquired on a FACS Canto II using Diva software (v6.2, BD Biosciences). At least 1x10^6^ PBMC were acquired, and analysis was conducted on at least 100.000 CD8^+^ events for each sample (34). **Supplementary Figures S2A–F** shows the gating strategy for TM^+^ cell identification.

**Table 3:** Characteristics of HLA-E-binding Mtb-derived peptides.

Peptide number	Amino acid sequence	Derived from	Amino acid position	Accession number^1^
34	VMTTVLATL	Rv1734c	42-50	P71992
44	RLPAKAPLL	Rv1484	53-61	P0A5Y6
55	VMATRRNVL	Rv1518	240-248	Q50590
62	RMPPLGHEL	Rv2997	470-478	O53244
68	VLRPGGHFL	Rv1523	251-259	Q50584

HLA-E binding motifs from Mtb-derived peptides were selected from (14). Amino acid sequences, originating proteins (Mtb H37Rv), as well as amino acid position and Swissprot accession numbers are indicated for the five peptides.

^1^UniProtKB/Swissprot accession number.

### *Ex vivo* expansion of HLA-E restricted CD8^+^ T cells by peptides or CyaA-LPE construct

PBMCs were expanded in 24 wells plates adding 5 μg/mL PHA (Remel, Thermo Fisher Scientific Inc., Santa Fe, NM), in IMDM added with 10% AB human serum and incubated at 37°C, 5% CO_2_ for three days, followed by the addition of 25 CU/mL IL-2 (Miltenyi Biotec). After six days of culture, a positive magnetic beads selection was used to sort CD8^+^ T cells (MACS, Miltenyi Biotec). With this method, more than >98% of purity was reached, as confirmed by FACS analysis (FACS Aria II, BD Biosciences). CD8^+^ T cells were put in culture with 10 μg/mL Mtb peptides or 1 μg/mL CyaA-LPE toxoid, 5ng/ml final concentration of both recombinant human IL-7 and IL-15 (Miltenyi Biotec) and allogeneic irradiated (30 Gy) APCs. On day 3 and day 5 of culture, the medium was replaced, adding complete medium with IL-2 (50 CU/ml) and IL-2 (100 CU/mL), respectively. On day 7, cultured CD8^+^ T cells were stained for HLA-E TMs and analysed by flow cytometry (35). **Supplementary Figures S2G, H** shows representative plots displaying the frequency of TMs^+^ CD8^+^ T cells after one week of culture with the pool of peptides or CyaA-LPE, respectively.

The HLA-E–restricted T-cell clone E#68-3 was generated as previously described and used as positive control. E#68-3 cells were incubated with human leukaemia monocytic cell line (THP-1) that had been stimulated for 72h with PMA (50 ng/mL final concentration) to induce differentiation towards macrophages, in the presence or absence of a pool of Mtb peptides or with CyaA-SPE or CyaA-LPE toxoids. After 24 h, the supernatants were collected and stored at -70° until testing. TNF-α levels were assessed by ELISA (R&D Systems).

### Intracellular cytokine staining and cytotoxic assay

The CD8^+^ T cells, expanded as mentioned above, were stimulated with a pool of Mtb peptides (10 µg/mL) in the presence of Brefeldin A (3μg/mL, Sigma-Aldrich) and monensin (1:1000, BioLegend, San Diego, CA) overnight at 37°C in 5% CO_2_. After incubation, stimulated cells were collected, washed with PBS/0.1% BSA, and stained with TMs at 37°C for 15 min. Then, cells were washed in PBS/0.1% BSA and stained for live/dead marker (Zombie dye, Biolegend), followed by staining (30 min, 4°C) for CD3 (PerCp-Cy5.5, Miltenyi Biotec, Clone REA613) and CD8 (PeCy7, Miltenyi Biotec, Clone BW135/80) surface expression, and finally fixing them using a fix/perm kit according to manufacturer’s protocol (BD Biosciences). For intracellular cytokine analysis, we used anti-human IFN-γ (APC, Biolegend, Clone B27), IL-10 (PEVio615, Miltenyi Biotec, clone MAb11) and IL-4 (FITC, Biolegend, Clone MP4-25) (36). The gating strategy is shown in **Supplementary Figure S3**.

At least 1x10^6^ events were acquired and analysed for all the samples with FlowJo software (version 9.9.6, Treestar Inc., Ashland). CD8^+^ T cells were sorted by using magnetic beads (Miltenyi Biotec) and were co-cultured with unpulsed or Mtb peptide-pulsed THP-1 cells, at an effector:target (E:T) ratio of 20:1 for 6 hrs. After incubation, the cells were stained with CD33 (APC, Miltenyi Biotec, clone REA775) used as threshold and Annexin-V-FLUOS staining kit to assess the cytotoxicity of target cells by Flow cytometry (**Supplementary Figure S4**).

### Statistical analysis

All data were analysed using Kruskal-Wallis or One way ANOVA test. P-values <0.05 were considered significant.

## Results

### The CyaA-SPE and CyaA-LPE constructs trigger human DC maturation *in vitro* and stimulate HLA-E-restricted and Mtb-specific CD8^+^ T cells

The CyaA-SPE and CyaA-LPE toxoids for HLA-E-restricted Mtb epitope delivery were constructed on the backbone of the adenylate cyclase toxoid B+D18 described previously (21), in which the residues 3-370 of the N-terminal AC domain of CyaA was replaced by synthetic oligo epitope sequences listed in **Table 2**. Using an *in vitro* assay, we verified that the CyaA-SPE and CyaA-LPE toxoids retained the capacity to deliver the SIINFEKL OVA reporter epitope into the cytosol of murine DCs for processing and MHC class I-restricted presentation to the OVA-specific CD8^+^ T cell line B3Z already at 0.2 nM concentration (**Supplementary Figure S1B**).

**Figure 1:**
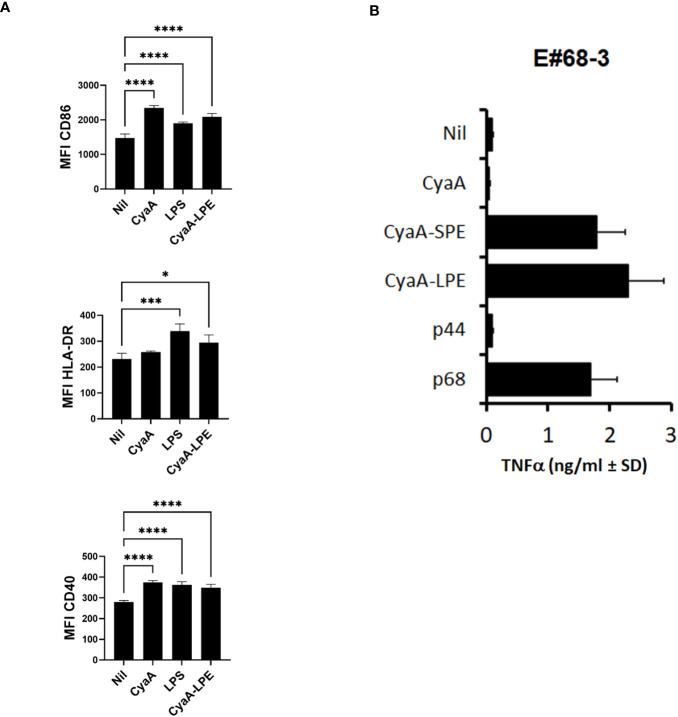
CyaA constructs induce DC maturation and activate HLA-E-restricted and Mtb peptide-specific CD8^+^ T cell clone. **(A)** the highly purified CyaA-LPE toxoid induces DC maturation (n=4). Shown is the surface expression of CD86, HLA-DR and CD40, measured as mean fluorescence intensity (MFI) ± standard deviation (SD). **(B)** TNF-α production of E#68-3 T cell clone stimulated with CyaA, CyaA-SPE, CyaA-LPE, peptide p44 or peptide p68. p-values were calculated using the Kruskal-Walli’s test, including multiple test corrections. *p<0.05, ***p<0.001, ****p<0.0001.

As the toxoids posess a poreforminfg activity that accounts for an inherent TLR-independent adjuvant activity (18), we next examined whether the CyaA-LPE toxoid would induce human DCs maturation. Human DCs were treated with endotoxin-free CyaA and 1 nM of CyaA-LPE toxoids for 24 hrs and analysed by flow cytometry, using CD86, CD40 and HLA-DR expression as maturation markers. As a positive control of DCs maturation stimulus, LPS was used at 50 ng/ml. As shown in **Figure 1A**, the highly purified endotoxin-free CyaA-LPE toxoid triggered an upregulation of CD86, HLA-DR and CD40 expression comparable to that induced by CyaA and by LPS. These results, hence, show that CyaA-LPE toxoid induce human DC maturation.

The ability of CyaA-SPE and CyaA-LPE toxoids to activate T-cell responses *in vitro* was compared to the stimulatory activity of the peptides without toxoids. To this aim, we used the CD8^+^ T cell clone E#68-3, which recognises the Mtb peptide p68 in association with HLA-E (37). We stimulated E#68-3 T cells with specific p68 peptide or with CyaA-SPE and CyaA-LPE in the presence of heterologous irradiated APCs. After 24 h, supernatants were collected, and TNF-α production was assessed by ELISA test as a readout. **Figure 1B** shows that a significant TNF-α production was obtained upon overnight incubation of E#68-3 T cells with both CyaA-SPE and CyaA-LPE toxoids, which was comparable or even superior to TNF-α production upon free p68 peptide stimulation. Notably, very little, if any, TNF-α production was observed when E#68-3 T cells were incubated with mock CyaA toxoid (CyaAM8a toxoid only containing OVA_257-276_ tag sequence) or with a different HLA-E-restricted peptide p44.

### The CyaA-LPE construct expands *in vitro* HLA-E-restricted and Mtb-specific CD8^+^ T cells

**Figure 2:**
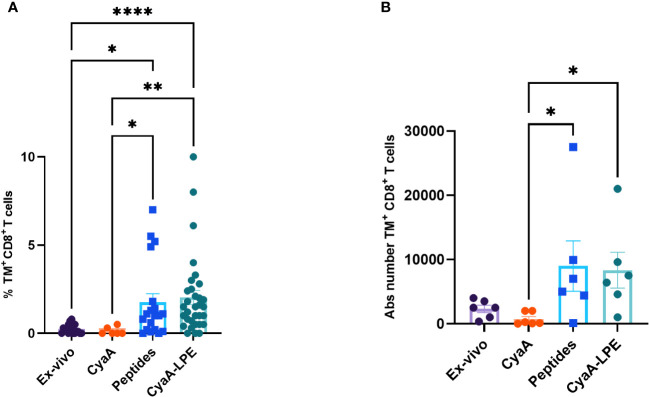
Comparison of frequency and absolute (Abs) numbers of HLA-E-restricted TM^+^ CD8^+^ T cells, *ex-vivo* and after *in vitro* expansion. PBMCs freshly collected *ex-vivo* and after *in vitro* expansion for 7 days with peptides or CyaA-LPE, were stained as above described. **(A)** The TM^+^ CD8^+^ T cells percentages are shown (n= 32). **(B)** The absolute number of TM^+^ CD8^+^ T cells is shown as calculated on six different subjects per each group. Each symbol represents one sample; bars represent mean values. p-values were calculated using the Kruskal-Walli’s test, including multiple test correction. *p<0.05, **p<0.01, ****p<0.0001.

The comparison of the TNF-α production by T cell stimulated with CyaA-SPE and CyaA-LPE toxoids did not show any statistical difference, but the CyaA-LPE construct performed better than SPE. We next examined the capacity of the CyaA-LPE toxoid to expand specific CD8^+^ T cells recognising the five HLA-E-restricted Mtb epitopes. Preliminary experiments (**Supplementary Figure S5**) showed that culturing PBMC, with mock CyaA toxoid did not result in an increase of specific TM^+^ CD8^+^ T cells compared to CyaA-LPE. As shown in **Figure 2A** and **Supplementary Figures S2G, H**, a 7-day culture of PBMCs with CyaA-LPE toxoid, caused an important expansion of specific CD8^+^ T lymphocytes (as detected using the 5 Mtb-peptides-HLA-E tetramers) which was slightly superior to that achieved by stimulation with a pool of free peptides (2.05% versus 1.75%). However, the differences were not statistically significant. TM^+^ CD8^+^ T cells, as absolute counts, were increased on average from 2,308 cells *ex vivo* (for 6 different PBMC samples) to 8,992 after expansion with a pool of the five peptides and 8,346 after expansion with CyaA-LPE, reaching an average expansion of 3.82-fold and 3.62-fold, respectively (**Figure 2B**).

The significantly huge number of TM^+^ CD8^+^ T cells obtained by the expansion protocol permitted us to study a detailed profile of their functional properties.

**Figure 3:**
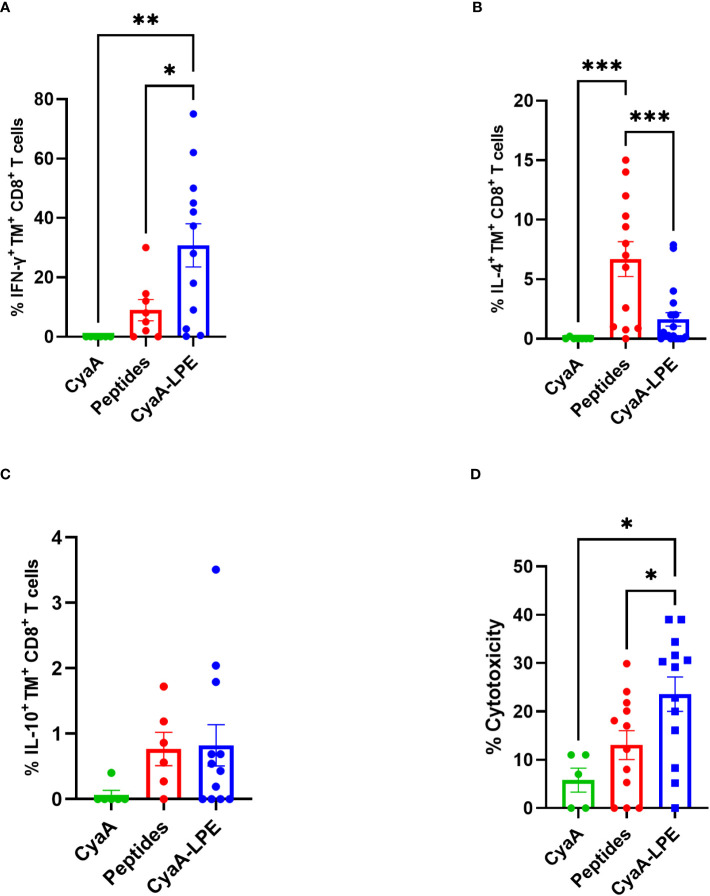
Cytokine production and cytotoxicity of peptide- or CyaA-LPE-expanded HLA-E TM^+^ CD8^+^ T cells. PBMCs (n=13) were expanded with peptides or CyaA-LPE and CD8^+^ T cells were sorted by magnetic bead. Cells were first stained with TMs and then with anti-CD3 and -CD8 mAbs for surface markers and with anti-IFN-γ, -IL4, and -IL10 mAbs for intracellular cytokines. **(A–C)** Frequency of TM^+^ CD8^+^ T cells producing IFN-γ, IL-4 or IL-10 upon peptide stimulation. **(D)** Cytotoxicity of CD8^+^ T cells, generated respectively with Mtb peptides and CyaA-LPE construct, against peptide-pulsed THP-1 macrophages, at an E:T ratio of 20:1. Each symbol represents one sample; bars represent mean values. The p-value was calculated using the One way ANOVA test with Holm-Sidak correction for multiple comparisons. *p<0.05, **p<0.01, ***p<0.001.

We evaluated the expression of cytokines by intracellular staining of HLA-E/Mtb TM^+^ CD8^+^ T cells upon short-term *in vitro* stimulation with a pool of the five Mtb peptides. **Figures 3A–C** shows the comparison between free peptide- or CyaA-LPE-expanded cells. In general, and in agreement with our previously published results, peptide-expanded TM^+^ CD8^+^ T cells produced both Tc1 (IFN-γ) and Tc2 (IL-4) cytokines as well as IL-10, upon Mtb peptide *in vitro* stimulation. Conversely, TM^+^ CD8^+^ T cells that had been expanded with CyaA-LPE appeared to have a more pronounced Tc1 cytokine profile. In fact, about 30% of TM^+^ CD8^+^ T cells on average were able to produce IFN-γ, while only less than 2% TM^+^ CD8^+^ T cells produced IL-4 and even less IL-10.

We next investigated the cytotoxic potential of HLA-E-restricted CD8^+^ T cells differently expanded by free peptides or CyaA-LPE construct. As shown in **Figure 3D**, the peptide-expanded CD8^+^ T cells exhibited a limited cytotoxic capacity towards THP-1 target cells pulsed with the five Mtb peptides. Conversely, *ex vivo* culture with the CyaA-LPE toxoid determined an increase of CD8^+^ T cells with a higher cytotoxicity potential towards THP-1 cells pulsed with the Mtb peptides. Thus, CyaA-LPE drives CD8^+^ T cells toward a more pronounced Tc1-type cytokine profile and improved cytotoxic activity to Mtb-derived antigens.

## Discussion

HLA-E has been the topic of numerous studies in recent years due to its low polymorphism and ability to present foreign peptides to CD8^+^ T cells and leading to an immune response. Additionally, HLA-E-restricted CD8^+^ T cells can be expanded *in vitro* and could serve as a subset of MHC-unrestricted T cells with great potential for adoptive cell therapy in infectious diseases. For these prerogatives, HLA-E represents a promising platform for the development of new vaccines that can directly activate the adaptive cell-mediated immune system in most of the world’s population, regardless of the HLA class Ia profile. In some cases, this promising role of HLA-E restricted CD8^+^ T cell memory subset to act against pathogens has been guaranteed as demonstrated, for instance, with a vaccine against SIV for NHP (17). However, many studies using peptides derived from Mtb have shown that the activation of HLA-E-restricted CD8^+^ T cells results in the production of cytokines like IL-4, IL-10 and TGF-β which are not protective against intracellular pathogens. Additionally, despite identifying immunogenic peptides, HLA-E-restricted CD8^+^ T cells have yielded heterogeneous results in terms of proliferation and cytotoxic or microbicidal activities in several experiments (7, 14, 35, 37). This issue can be overcome by effectively redirecting HLA-E-restricted CD8^+^ T cells towards a Tc1 profile.

Our results show that delivery of the HLA-E-binding Mtb epitopes by CyaA recombinant toxoids induces expansion of Mtb-specific and HLA-E-restricted CD8^+^ T cells in PBMCs obtained from TB patients. A significant expansion of HLA-E-restricted CD8^+^ T cells was achieved by CyaA recombinant toxoid carrying two or five distinct Mtb epitopes, which was comparable to expansion achieved upon stimulation with corresponding free peptides.

Importantly, however, both CyaA recombinant toxoids activate HLA-E-restricted specific CD8^+^ T cells at a low concentration (1 µg/mL), at which concentration-free peptides induce minimal activation, if any. In fact, free peptides are typically used to activate Mtb-specific CD8^+^ T cells at a concentration of 10 µg/mL (14, 38). In addition, while CD8^+^ T cell activation often requires adjuvant molecules to maximise the extent of the activation threshold, delivery of peptides by CyaA toxoids is achieved without the help of adjuvant. This is most likely due to the unique adjuvanticity of the CyaA toxoid, which specifically targets and permeabilises DCs expressing CD11b/CD18 and promotes inflammasome activation and IL-1β production (39).

The CyaA toxoid plays a dual role in the immune system. In addition to deliver epitopes into the DCs’ cytosol for processing and presentation by MHC class I molecules to CD8^+^ T cells, it also enters the MHC-class II presentation pathway to activate CD4^+^ T cells (19). This is relevant to HLA-E antigen presentation since the pathways responsible for peptide loading onto HLA-E and the cellular compartments where this occurs remain largely unknown.

The most striking finding of our study was that expansion of Mtb-specific and HLA-E-restricted CD8^+^ T cells by CyaA-LPE toxoid was paralleled by the acquisition of a predominant Tc1 cytokine profile with a significant increase of IFN-γ production upon *in vitro* stimulation of expanded cells with specific Mtb peptides. This contrasts with the mixed Tc1/Tc2 profile of CD8^+^ T cells expanded with free peptides, as shown here and in our previous studies (7, 14, 35, 37). Moreover, the CyaA-LPE-expanded CD8^+^ T cells displayed a cytotoxic potential towards peptide-pulsed macrophages significantly superior to CD8^+^ T cells expanded by the free peptide pool.

This finding agrees with previous studies showing that CyaA toxoids shift the polarisation of the immune response from a typical Th2 type to a mixed Th1/Th2 type of response (27). The immunogenic properties of the *Bordetella pertussis* toxoid are responsible for this ability. When the CyaA toxoid binds to the CD11b/CD18 complex on APCs such as DCs, it creates pores on the plasma membrane and activates a pathway that mainly induces the cell to increase the expression of costimulatory molecules such as CD80, CD86 and CD40 (18). In a separate study in mouse model, the administration of another CyaA-based construct, the CyaA-E5-Tat which brings epitopes from HIV-1 Tat protein, induces a Tc1 in mice, CD8^+^ T cell activation was observed after administering CyaA-OVA. This led to the expansion of responding cells and their cytokine production. The study also demonstrated that CyaA toxoid induced DCs maturation which was TLRs-independent and relied on p38/JNK and MAPK signaling (18).

In conclusion, the present results show that CyaA toxoids delivering inserted Mtb epitopes efficiently expand HLA-E-restricted and Mtb-specific human CD8^+^ cytotoxic T cells present in PBMC samples from TB patients. Hence, the delivery of Mtb peptides by the CyaA toxoid represents a particularly effective method in inducing the Tc1 response capable of counteracting Mtb infection. Furthermore, the construct we evaluated could be a component of a subunit vaccine more advantageous in production and storage costs and, therefore, more usable than an mRNA vaccine in low-income countries where TB is more widespread. Nineteen vaccines against Mtb are under clinical trial (40), but no mRNA vaccine is included. There is only one report of a mRNA vaccine against Mtb encoding the MPT83 antigen formulated in 2004, but it was found poorly immunogenic in a mouse model (41).

The value of the HLA-E molecule in protection against mycobacterial infection has been highlighted in other studies. Mtb-infected mice deficient in HLA-E homolog molecule Qa-1 have a high mortality rate and increased systemic Mtb burden (42). Finally, our own studies have shown that HLA-E-restricted CD8^+^ T cells have increased cytotoxicity and antimicrobial function against macrophages infected with Mtb and HIV (7), suggesting that an HLA-E-based platform to Mtb vaccination could have a significant impact in areas where HIV and Mtb coinfection is prevalent.

## Data availability statement

The raw data supporting the conclusions of this article will be made available by the authors, without undue reservation.

## Ethics statement

The studies involving humans were approved by Institutional Review Board of Palermo University Hospital (approval number 13/2013). The studies were conducted in accordance with the local legislation and institutional requirements. The participants provided their written informed consent to participate in this study. Ethical approval was not required for the studies on animals in accordance with the local legislation and institutional requirements because only commercially available established cell lines were used.

## Author contributions

GDB: Data curation, Formal Analysis, Methodology, Software, Writing – original draft. MPLM: Formal Analysis, Methodology, Writing – original draft, Investigation, Writing – review & editing. PDC: Methodology, Writing – review & editing, Resources. OS: Methodology, Writing – review & editing. IL: Methodology, Writing – review & editing, Validation, Visualization. NC: Visualization, Supervision, Writing – review & editing. PS: Methodology, Resources, Writing – review & editing. FD: Funding acquisition, Methodology, Project administration, Resources, Supervision, Writing – review & editing.
