# Supplementary material for: Delivery of Mycobacterium tuberculosis epitopes by Bordetella pertussis adenylate cyclase toxoid expands HLA-E-restricted cytotoxic CD8+ T cells
Source: Front Immunol. 2023 Dec 1;14:1289212. doi: 10.3389/fimmu.2023.1289212 (PMC10722248; doi:10.3389/fimmu.2023.1289212)
Supplement: Supplementary file 1 [file DataSheet_1.docx]

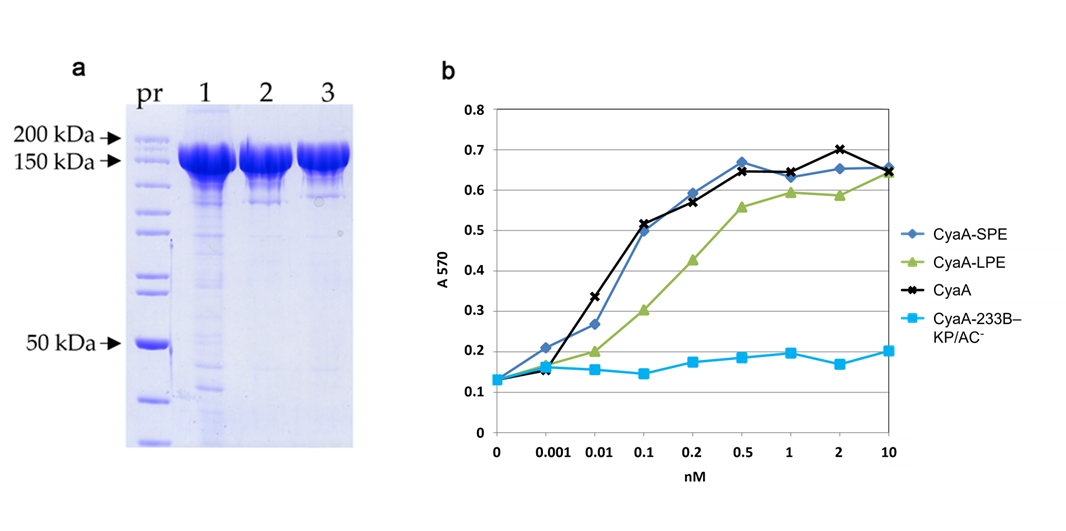

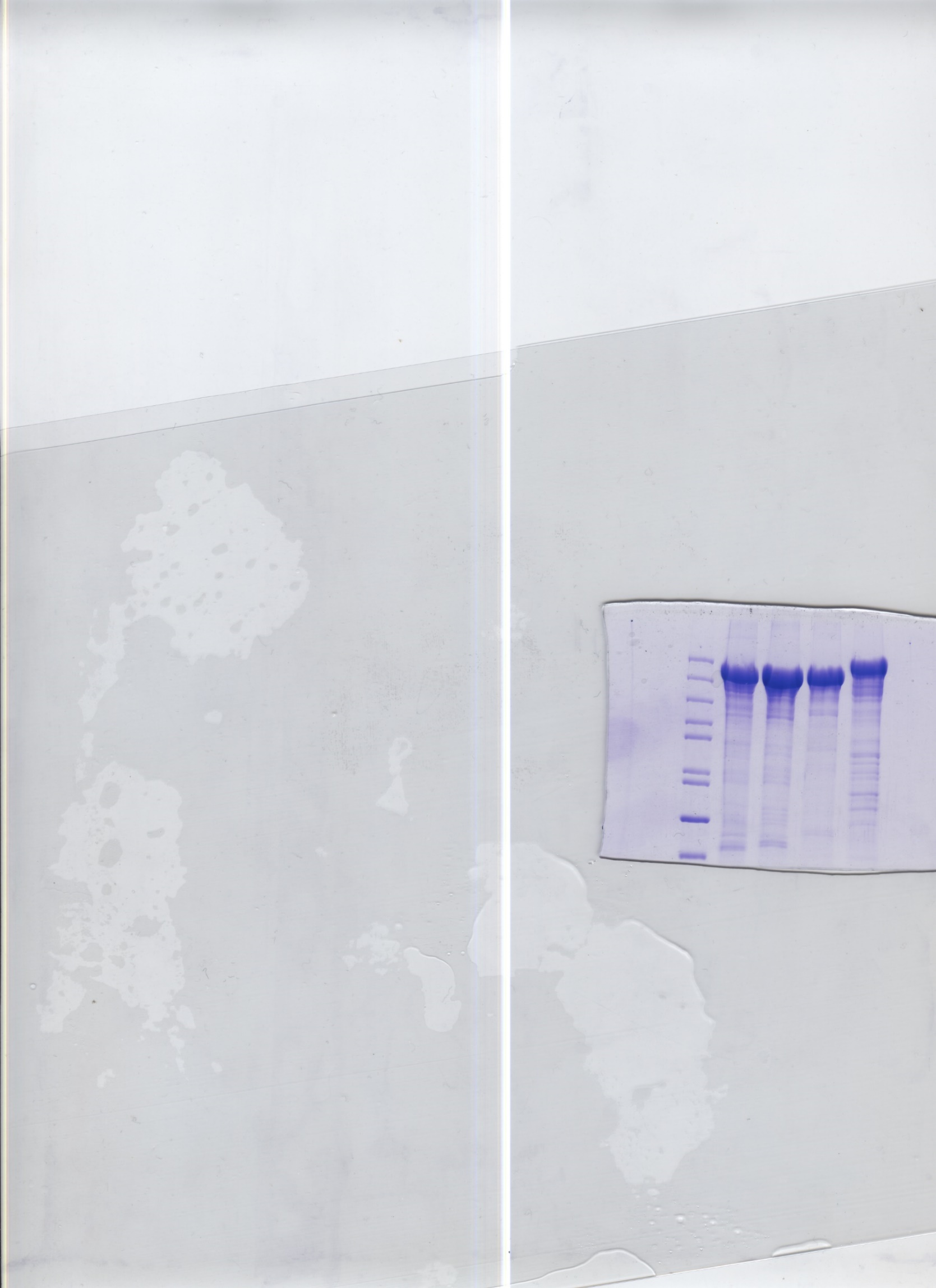


200 kDa

50 kDa

150 kDa

pr

1

2

3

4

**Supplementary Figure S1.** Purification and antigen-presenting capacity of CyaA antigenic constructs.

1. The CyaA toxoid samples were separated by 7.5% SDS-PAGE and visualised by Coomassie blue staining. pr- page ruler (Thermo Scientific™ PageRuler™ Unstained Protein Ladder; 26614); 1 = CyaA(Cya-Δ3-370-OVA_257-276_) control toxoid; 2 = CyaA-SPE; 3 = CyaA-LPE; 4 = CyaA 223B-KP/AC^-^.
2. The capacity to translocate the AC domain into the cytosol is required for efficient delivery of the OVA_257-264_ epitope for presentation on MHC class I molecules. DC2.4 cells were pulsed with the indicated concentrations of toxoids for 4 h. After being washed with PBS, the DC2.4 cells were further cultured for 18 h with the B3Z CD8 T-hybridoma cells that selectively recognize cell surface presented complexes of the H-2K^b^ MHC class I molecules with bound OVA_257-264_ peptide (SIINFEKL). As a positive control the CyaA toxoid carrying the OVA_257-276_ epitope was used and as negative control, the CyaA-233B–KP/AC^-^ that carries the OVA_257-264_ epitope SIINFEKL inserted between residues 232 and 233 of the full-length CyaA-AC^-^ toxoid [21] and is still capable of bindingCD11b/CD18 receptor of antigen presenting cells, but is unable to deliver the AC domain with the inserted epitopes into their cytosol for processing and MHC class I-restricted antigenic presentation due to a combination of the E570K+E581P substitution in the translocator domain of the CyaA-AC^-^ toxoid [21, 29]. Antigenic stimulation of B3Z cells was assayed as the amount of accumulated β-galactosidase expressed from the *lacZ* reporter gene under the control of the interleukin 2 (IL-2) promoter NF-AT elements that are activated upon TCR recognition of the OVA257-264 peptide (SIINFEKL) on the murine H-2K^b^ MHC class I molecules [30].
