# Supplementary material for: Delivery of Mycobacterium tuberculosis epitopes by Bordetella pertussis adenylate cyclase toxoid expands HLA-E-restricted cytotoxic CD8+ T cells
Source: Front Immunol. 2023 Dec 1;14:1289212. doi: 10.3389/fimmu.2023.1289212 (PMC10722248; doi:10.3389/fimmu.2023.1289212)
Supplement: Supplementary file 2 [file DataSheet_2.docx]

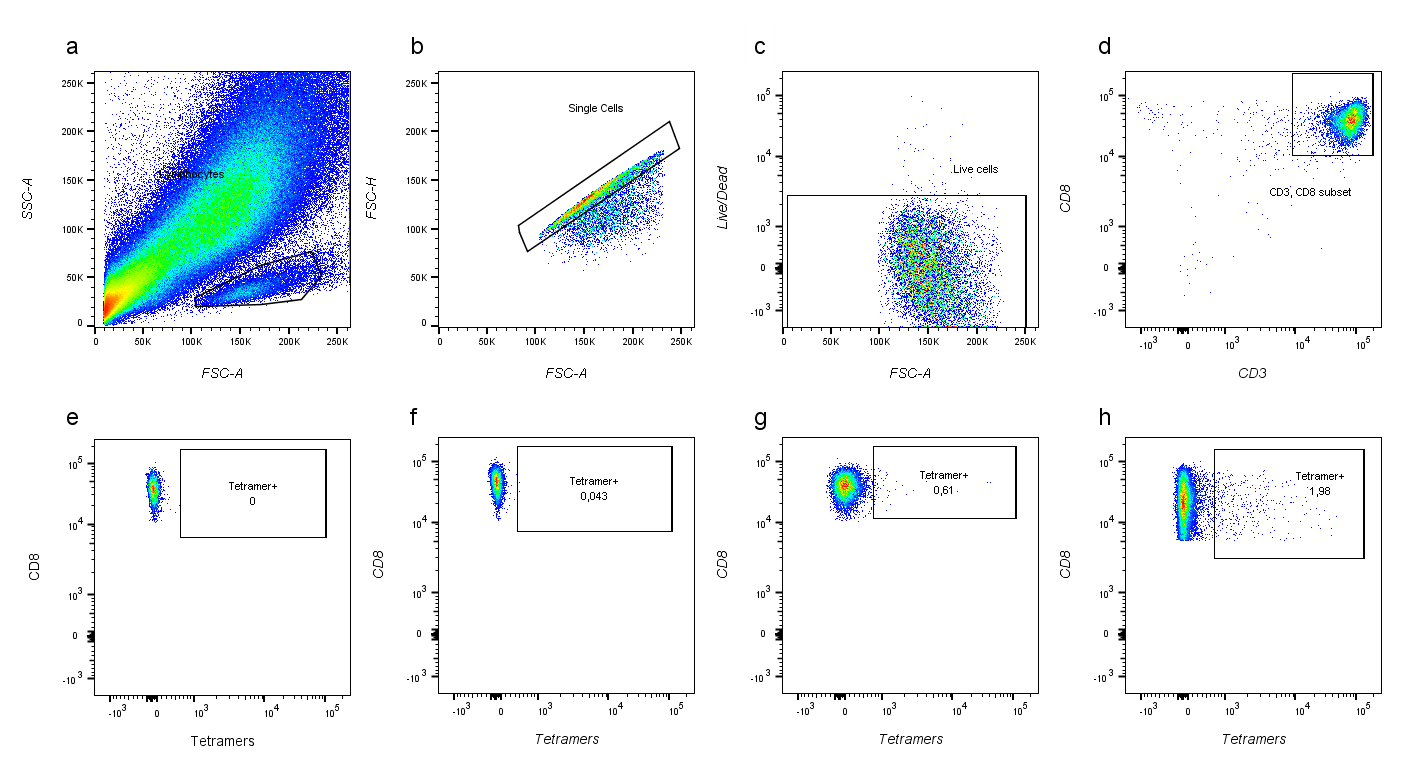


**Supplementary Figure S2**. Representative gating strategy of CD8^+^ TM^+^ cells *ex vivo* and after coculture with the pool of peptides or CyaA-LPE.

Shown is an initial gate on lymphocytes (a) and single cells (b), followed by gating on live cells (c) and CD3 and CD8 double positive cells (d). Then, gating was as performed on CD8^+^ and TM^+^ cells. e) Negative control (i.e. no TM staining). f, g and h) Show TM^+^ CD8^+^ T cells *ex vivo*, after one week of *in vitro* culture with peptides or CyaA-LPE, respectively.
