# Supplementary material for: Delivery of Mycobacterium tuberculosis epitopes by Bordetella pertussis adenylate cyclase toxoid expands HLA-E-restricted cytotoxic CD8+ T cells
Source: Front Immunol. 2023 Dec 1;14:1289212. doi: 10.3389/fimmu.2023.1289212 (PMC10722248; doi:10.3389/fimmu.2023.1289212)
Supplement: Supplementary file 3 [file DataSheet_3.docx]

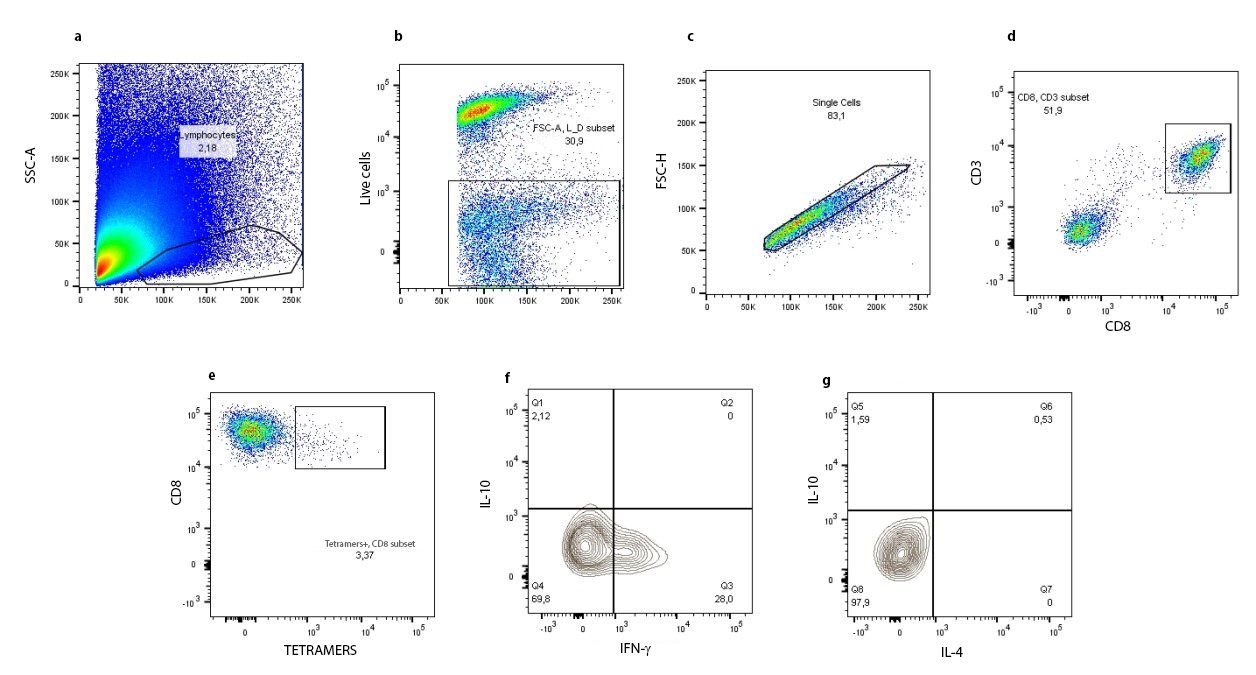


**Supplementary Figure S3**.  Representative gating strategy to assess the production of cytokines by T CD8^+^ TM^+^ cells after one week of culture with peptides or CyaA-LPE.

Shown is sequential gating on live lymphocytes (**a,b**), single cells (**c**), CD3^+^ CD8^+^ T cells (**d**), CD8^+^ TM^+^cells (**e**) and CD8^+^ TM^+^T cells positive for IFN-γ, IL-10 and IL-4 (**f, g**).
