# Supplementary material for: Delivery of Mycobacterium tuberculosis epitopes by Bordetella pertussis adenylate cyclase toxoid expands HLA-E-restricted cytotoxic CD8+ T cells
Source: Front Immunol. 2023 Dec 1;14:1289212. doi: 10.3389/fimmu.2023.1289212 (PMC10722248; doi:10.3389/fimmu.2023.1289212)
Supplement: Supplementary file 4 [file DataSheet_4.docx]

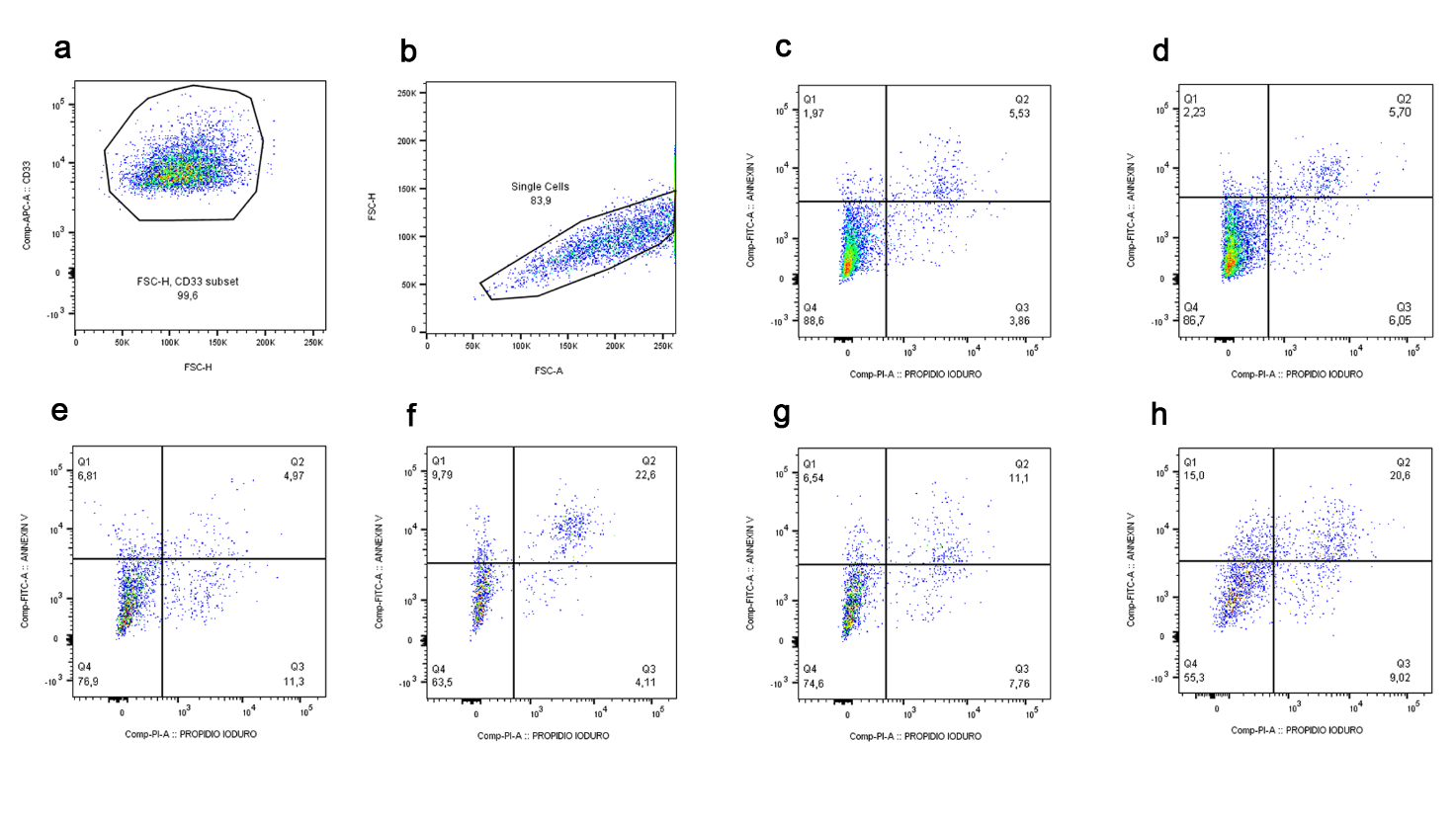


**Supplementary Figure S4**. Annexin V/Propide iodure (PI) cytotoxicity assay.

Identification of THP1-derived macrophages (**a, b**); unpulsed macrophages (**c**); peptides-pulsed macrophages (**d**); unpulsed macrophages cultured with peptide-specific CD8^+^ T cell line (**e**); peptides-pulsed macrophages cultured with peptide-specific CD8^+^ T cell line (**f**); unpulsed macrophages cultured with CyaA-LPE CD8^+^ T cell line (**g**); peptides-pulsed macrophages cultured with CyaA-LPE CD8^+^ T cell line (**h**).
