# Supplementary material for: Delivery of Mycobacterium tuberculosis epitopes by Bordetella pertussis adenylate cyclase toxoid expands HLA-E-restricted cytotoxic CD8+ T cells
Source: Front Immunol. 2023 Dec 1;14:1289212. doi: 10.3389/fimmu.2023.1289212 (PMC10722248; doi:10.3389/fimmu.2023.1289212)
Supplement: Supplementary file 5 [file DataSheet_5.docx]

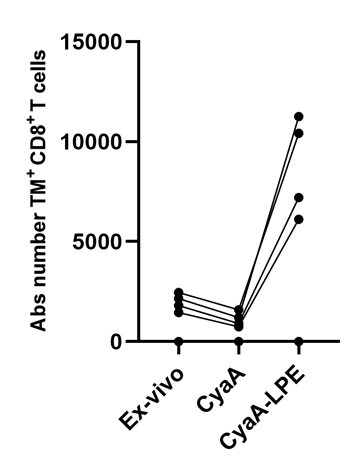


**Supplementary Figure S5.** Absolute (Abs) numbers of HLA-E-restricted TM^+^ CD8^+^ T cells, *ex-vivo* and after *in vitro* expansion.

PBMCs freshly collected *ex-vivo* and after *in vitro* expansion for 7 days with CyaA or CyaA-LPE, were stained as described in Materials and Methods.  Shown is the absolute number of TM^+^ CD8^+^ T cells.
